# Enhancing the antibacterial ability of *Ligilactobacillus salivarius* through ARTP mutagenesis breeding: an effective strategy to improve its probiotic properties

**DOI:** 10.3389/fmicb.2025.1595651

**Published:** 2025-07-21

**Authors:** Hetian Zhou, Yunping Du, Guolian Yu, Wei Liu, Juanjuan Zeng, Bin Wang, Maojin Chen, Yujue Wang, Wenbo Zhang, Xiaona Wei

**Affiliations:** ^1^School of Life Sciences, Zhengzhou University, Zhengzhou, China; ^2^Wen’s Group Academy, Wens Foodstuff Group Co., Ltd., Xinxing, China; ^3^Jiangxi Biotech Vocational College, Nanchang, China; ^4^College of Animal Science and Technology, Jiangxi AgriculturalUniversity, Nanchang, China

**Keywords:** *Ligilactobacillus salivarius*, antibacterial ability, probiotic properties characteristics, ARTP mutagenesis, screen strains

## Abstract

**Introduction:**

*Ligilactobacillus salivarius* (*L. salivarius*), a well-characterized probiotic species with established safety and functional efficacy, has been widely applied in poultry production for decades. Its probiotic attributes primarily encompass inhibiting pathogenic bacterial proliferation, regulating host intestinal microbiota, and modulating immune responses to enhance animal health. Given the substantial variability in biological and probiotic characteristics among different *L. salivarius* strains, identifying optimal strains with enhanced probiotic efficacy typically requires extensive *in vivo* evaluations.

**Methods:**

In this study, we employed atmospheric and room temperature plasma (ARTP) mutagenesis to improve the antibacterial activity of the isolated D428 strain. Following ARTP mutagenesis and positively screened for its antibacterial ability, the mutant strain 30s-37 was obtained. By comparing the effects of the parental (D428) and mutant (30s-37) strains on broiler growth performance and intestinal microbiota, their probiotic properties performance was evaluated.

**Results:**

The results indicated that the use of *L. salivarius* strains improved the growth performance and increased the richness and diversity of cecal probiotic microbial communities, with the mutagenized strain 30s-37 exhibiting more pronounced effects.

**Discussion:**

These findings underscore mutagenesis breeding as an effective strategy for probiotic strain optimization, justifying its expanded application in future strain development programs.

## Introduction

*Lactobacillus*, an important microbial population in the gastrointestinal tract of humans and animals (Djadouni and Kihal, 2012; Todorov, 2009), is considered a potential probiotic due to its ability to regulate gut homeostasis (Gasbarrini et al., 2016; Tsuda and Miyamoto, 2010), prevent pathogenic attachment (Parada et al., 2007; Pourabedin and Zhao, 2015; Zacharof and Lovitt, 2012), and stimulate the host immune system (Yang et al., 2024; Zhang et al., 2016). *Ligilactobacillus salivarius* (*L. salivarius*) is commonly found in the oral cavity, digestive tract, and feces of humans and animals, exhibiting excellent probiotic properties and biological characteristics (Raftis et al., 2011; Long et al., 2018). It possesses properties such as acid resistance, bile salt resistance, and high extracellular polysaccharide production, and demonstrates superior adhesion and colonization abilities on intestinal epithelial cells (Neville and O'Toole, 2010; Messaoudi et al., 2013). *L. salivarius* strains have been applied in poultry farming for years (Pascual et al., 1999). Shokryazdan *et al.* reported that feeding broilers with a mixture of *L. salivarius* strains (CI1, CI2, and CI3) increased body weight, reduced total cholesterol, LDL cholesterol, and triglycerides, increased the number of beneficial microorganisms, and decreased harmful microorganisms (Shokryazdan et al., 2017). Wang et al. found that administering *L. salivarius* strains to chickens improved host growth performance (e.g., body weight and shank length) and mitigated organ damage caused by *E. coli* O78 and heat stress (Wang et al., 2020). These findings indicate that *L. salivarius* strains can enhance poultry growth performance and improve host health. Sornplang et al. reported that *L. salivarius* strains L61 and L55 supplements stimulated heterophil phagocytic activity in broilers infected with *Salmonella* (Sornplang et al., 2015). Like most beneficial microorganisms, *L. salivarius* strains can improve poultry growth performance and health. For instance, Xu et al. used *L. salivarius* strain CML352 to regulate the intestinal microbiota of laying hens, improving intestinal health and egg quality in late-phase hens (Xu et al., 2022). Saint-Cyr et al. found that *L. salivarius* strains SMXD51 exhibited anti-*Campylobacter* activity *in vivo* and could partially prevent *Campylobacter*’s impact on the poultry gut microbiota (Saint-Cyr et al., 2017).

Atmospheric and room temperature plasma (ARTP) mutation technology is an innovative and efficient technique for microbial breeding. It is characterized by high mutation efficiency, biosafety, operational simplicity, rapid mutagenesis, and high-throughput library generation. (Dong et al., 2010; Li et al., 2012). Therefore, it holds significant potential in the field of microbial mutation breeding. ARTP mutation is widely applicable to both eukaryotes (including yeast, fungi, algae, higher fungi, plant seedlings, callus tissues, seeds, or protoplasts) and prokaryotes. Wang et al. were the first to use ARTP mutation technology on the spores of *Streptomyces avermitilis* for 3 min (Wang et al., 2010). A lethality rate of 98.2%, a total mutation rate exceeding 30%, and a positive mutation rate of approximately 21% were achieved. After screening and cultivation, a stable mutant was obtained, exhibiting more than a 40% increase in avermectin B1a production and an 18% total yield increase compared to the wild strain. Hua et al. utilized ARTP mutation to enhance the salt tolerance of *Enterobacter cloacae*, enabling its growth in high-salinity soil environments and increasing the total petroleum hydrocarbon degradation rate by 2.5 times (Hua et al., 2010). Zhao et al. applied ARTP mutation breeding technology, resulting in a mutant strain with DHA production increased 1.8 times after screening. During the scale-up process, the average DHA production reached 14.0 g/L with the addition of Fe^2+^ (Zhao et al., 2018).

In animal husbandry, the screening of probiotic strains with optimal functional traits relies heavily on laborious and time-consuming *in vivo* trials, which significantly impedes the efficiency of strain development. The probiotic effects of these microorganisms are primarily attributed to their capabilities of gut colonization, secretion of antibacterial compounds, and production of beneficial metabolites such as lactic acid and butyric acid. Therefore, we speculate that enhancing specific characteristics of probiotic properties through *in vitro* mutagenesis, such as improving antibacterial activity or increasing butyric acid production, may improve the probiotic properties of the strains and enhance screening efficiency. In this study, we selected *L. salivarius* D428 strain as the object to evaluate the probiotic properties after ARTP mutagenesis, positive selection for antibacterial ability and *in vivo* evaluation in broiler chickens to evaluate their probiotic properties after mutagenesis, to assess the effectiveness of enhancing the probiotic properties of probiotics through *in vitro* mutagenesis.

## Materials and methods

### Isolation and identification of *Ligilactobacillus salivarius* strains

The cecum content from yellow feather broilers was collected in Yunfu, Guangdong, China. Briefly, 10 g of cecum content was added into 90 mL sterile Phosphate buffer saline (PBS) with glass beads aseptically and shaken at 200 r/ h for 1 h at 37°C. A sample was taken at a 1:9 ratio and added to MRS broth (BaseBio, China), then incubated at 37°C for 24 h statically. Subsequently, diluted cultures were spread on MRS agar plates containing CaCO_3_ (Wright and Klaenhammer, 1981). After 48 h of incubation at 37°C, single colonies with transparent calcium-dissolving zones were picked and streaked on MRS plates until single, uniform, pure colonies were obtained. Store the pure colonies at 4°C for future use. The isolated strains were identified using Gram staining and 16S rDNA sequencing. According to the instructions received, microbial genomic DNA was extracted using the TIANamp Bacteria DNA Kit (TIANGEN, China). The 16S rDNA gene was amplified using universal PCR primers (5’-GGTTACCTTGTTACGACTT-3′, 5’-AGAGTTTGATCMTGGCTCAG-3′). The 30 μl PCR reaction system included 2 μl DNA template, 15 μl 2 × Ex Taq PCR mix, 0.3 μM of each primer, and ddH_2_O. The PCR amplification procedure was as follows: 95°C for 3 min, followed by 35 cycles of denaturation at 95°C for 30 s, primer annealing at 53°C for 30 s, elongation at 72°C for 2 min, and final extension at 72°C for 5 min. The PCR products were sequenced at Sangon Biotech and analyzed using nucleotide BLAST on the NCBI website. The molecular phylogenetic tree was constructed using MEGA (MEGA-X) software with the Neighbor Joining method.

### *In vitro* antibacterial assay of *Ligilactobacillus salivarius* strains

The Oxford cup method was utilized to perform the *in vitro* inhibition assay based on the agar diffusion principle. Briefly, 100 μl of each indicator bacterium culture, which had been grown to the logarithmic phase, was added to 100 mL of sterilized LB solid medium cooled to 45°C. After thorough mixing, 16 mL of the bacterial-medium mixture was poured into sterile Petri dishes. Once the medium solidified, sterile Oxford cups were evenly placed on the surface. Subsequently, 200 μl of the *L. salivarius* strain suspension was added to each Oxford cup. To allow complete diffusion of the suspension into the medium, the plates were first incubated at 4°C for 4 h. They were then transferred to a 37°C incubator for 10–12 h of cultivation. Finally, the diameter of the resulting inhibition zones was measured using a vernier caliper. Indicator bacteria employed in this study included *Escherichia coli* (*E. coli*), *Staphylococcus aureus* (*S. aureus*), and *Salmonella pullorum* (*S. pullorum*), which were previously isolated and identified in our laboratory.

### Growth curve and acid production

The *L. salivarius* strains exhibiting the highest *in vitro* antibacterial activity were selected for cultivation. These strains were inoculated into 100 mL MRS broth at a 1% inoculum ratio and statically incubated at 37°C. At 2-h intervals, 1 mL aliquots of the bacterial suspension were collected to measure optical density at 600 nm (OD_600_), with data plotted to generate a growth curve. Concurrently, the pH of each sample was measured using a laboratory pH meter, and results were similarly plotted to create a pH change curve.

### Stress tolerance

#### Bile salt tolerance

Activated *L. salivarius* strains were inoculated into MRS broth supplemented with 0.1, 0.2, 0.3, 0.4, and 0.5% chicken bile salt at a 2% inoculation ratio, with bile salt-free MRS broth as the control. Cultures were incubated statically at 37°C for 6 h. Post-incubation, the bacterial suspensions from each bile salt concentration were subjected to 10-fold serial dilutions, and 100 μl of the 10^−5^ and 10^−6^ dilutions were spread on MRS agar plates. The plates were incubated at 37°C for 24 h, after which colony-forming units (CFUs) were enumerated. Strain survival rates were calculated as follows:

$$\mathrm{Survival rate}\;\left(\%\right)=\frac{\mathrm{CFUs in treatment group}}{\mathrm{CFUs in control group}}\times 100\%$$

Each treatment was performed in triplicate.

#### Tolerance to stimulated gastric and intestinal fluids

Activated *L. salivarius* strains (10% v/v) were inoculated into artificial gastric and intestinal fluids, respectively, followed by static incubation at 37°C. Samples were collected at 0 and 2 h, centrifuged at 5,000 × *g* for 5 min, and the supernatants were discarded. Pellets were resuspended in physiological saline solution (PBS), subjected to 10-fold gradient dilution, and 100 μl of the 10^−5^, 10^−6^, and 10^−7^ dilutions were spread on MRS agar plates. Plates were incubated statically at 37°C for 24 h, after which colonies were counted. Tolerance to artificial gastric and intestinal fluids was determined using the formula below:

$$\mathrm{Survival rate}\;\left(\%\right)=\frac{\mathrm{CFUs after}\;2\;\mathrm{hours of treatment}}{\mathrm{CFUs}\;\mathrm{at}\;0\;\mathrm{hours}}\times 100\%$$

All experiments were performed in triplicate.

### ARTP mutagenesis of *Ligilactobacillus salivarius* strain D428

Following biological characterization, *L. salivarius* strain D428, which exhibited the optimal growth rate, acid-production capacity, and tolerance to bile and gastrointestinal fluids, was subjected to ARTP mutagenesis to enhance its *in vitro* antibacterial activity. Briefly, activated *L. salivarius* strain D428 was cultured in fresh MRS broth for 6 h. After being washed with sterile ultrapure water, the bacteria were resuspended in sterile 10% glycerol (1:1 v/v) to achieve a cell density of 10^6^–10^8^ CFU/mL. A 10 μl aliquot of the bacterial suspension was evenly spread onto the sterile metal slide and placed into the ARTP mutagenesis chamber. Treatment parameters followed the manufacturer’s protocol: gas flow rate at 10 SLM, power at 120 W, and treatment times of 0, 15, 30, 45, 60, 90, and 120 s. After mutagenesis, treated samples were thoroughly mixed for 1 min, tenfold serially diluted, and plated on MRS agar for colony enumeration. Each treatment was performed in triplicate. The colonies derived from different mutagenesis times were screened for antibacterial activity using the Oxford cup method. The lethality rate and positive mutation rate were calculated as follows:

$$\begin{array}{l} \mathrm{Lethality rate}\;\left(\%\right)=\\ \frac{\mathrm{Untreated viable count}-\mathrm{Treated vival count}}{\mathrm{Untreated viable count}}\times 100\% \end{array}$$

A lethality curve was constructed based on these calculations.

### Screening of mutagenized *Ligilactobacillus salivarius* strains

The Oxford cup method was used to screen the *in vitro* antibacterial activity of randomly selected individual mutagenized *Lactobacillus* strains. Mutations with enhanced antibacterial activity are defined as positive mutations as follows:

$$\begin{array}{l} \;\mathrm{Positive mutation rate}\;\left(\%\right)=\\ \frac{\mathrm{Number of enhanced inhibitory potency mutants}}{\mathrm{Total vival bacteria for bacteriostatic titer determine}}\times 100\%. \end{array}$$

The strains with high antibacterial efficiency were subjected to continuous passage, and *in vitro* antibacterial tests were conducted every two passages to verify the stability of the antibacterial ability of the mutagenized *Lactobacillus*.

### Animal experiments

The animal study protocol was approved by the ethical guidelines and the animal experimental safety review system of Jiangxi Agricultural University ([2018]30). One-day-old Qingyuan Ma cockerels were randomly divided into three groups. Group A’s diet was supplemented with *L. salivarius* strain D428. Group B’s diet was supplemented with mutant 30s-37. Group C was set as the control group. Each group consisted of 75 chickens. The duration of the experiment was 71 days. The bacterial counts for Groups A and B were both 5 × 10^5^ CFU/g and mixed with feeds. The feeding and management of each group were completely the same. Feeding, water provision, cleaning, disinfection, and disease prevention were strictly carried out following the standards of Wens Foodstuff Group Co., Ltd. During the brooding period, heat lamps was used to regulate the temperature at 35°C in the first week and then gradually decreased by 2–3°C every week until reaching the room temperature. There was full-day lighting during the brooding period. At the end of the experiment, the chickens were weighed. Feed control started the night before weighing, but drinking water was not restricted. Record the average feed intake, the average initial weight at the beginning of the test, the average final weight at the end of the test, the average daily weight gain, the feed conversion ratio, the mortality and culling rate, as well as the occurrence of other diseases and the corresponding treatment measures, etc.

### 16S rDNA amplicon sequencing and analysis

After the experiment, three chickens from each group were randomly selected and slaughtered. All animals were euthanized by asphyxiation with CO_2_ as per the ethical guidelines and animal experimental safety review system of Jiangxi Agricultural University ([2018]30). Cecum contents were collected under sterile conditions in 10 mL centrifuge tubes, promptly labeled, and stored in liquid nitrogen. The samples were then sent to the Magigene Biotechnology Co., Ltd. (Guangzhou, China) for cecal microbiota diversity analysis. The procedure was as follows: DNA was extracted from qualified samples and checked for quality. The V4-V5 region of 16S rDNA was specifically amplified, and the purified PCR products were used for library construction and subjected to high-throughput sequencing. Sequence analysis and species annotation were performed to determine the intestinal microbial composition. Comparative analysis of community composition and differences at the taxonomic level between samples was conducted to assess variability.

### Statistical analysis

All measurements were repeated independently in triplicate, and results were expressed as mean ± standard deviation (SD). Data obtained were statistically analyzed using SPSS Software (V21.0, IBM). Significance level was expressed by *p*-value, and differences were considered statistically significant at *p* < 0.05.

## Results

### Isolation and identification of *Ligilactobacillus salivarius* strains D428

**Figure 1:**
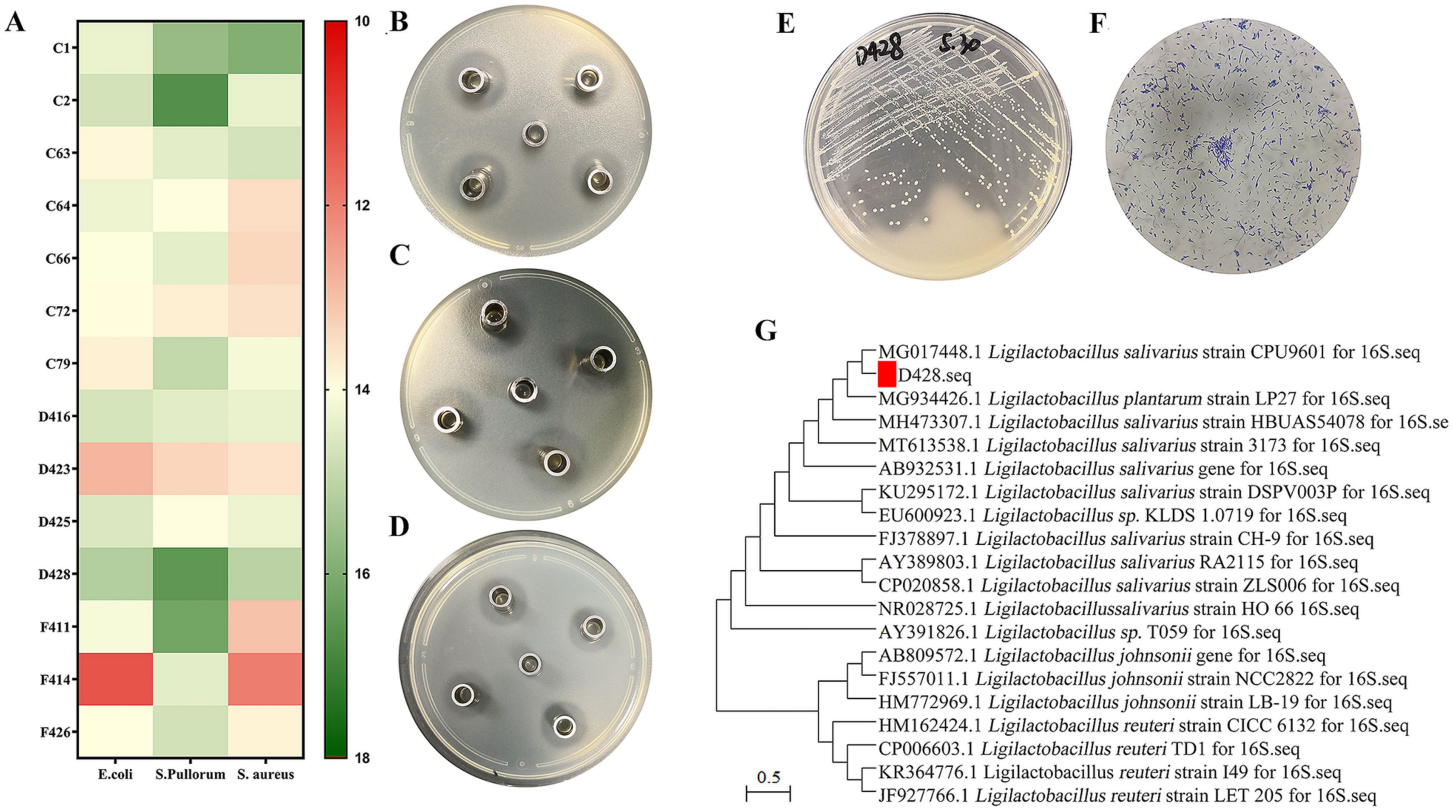
Isolation and identification of *L. salivarius* strains D428. **(A)** Among 64 isolates, fourteen lactic acid bacterial strains exhibited stable and potent inhibitory activity against *Salmonella, Escherichia coli,* and *Staphylococcus aureus*. **(B–D)** The Oxford cup method was used to detect the anti*-Salmonella,* anti*-Escherichia coli,* and anti*-Staphylococcus aureus* activity, respectively. **(E)** The colony morphology, **(F)** the Gram staining, and **(G)** the molecular phylogenetic tree of 16SrDNA of the isolated strain D428.

A total of 64 isolates displaying distinct calcium-dissolution halos and various colony morphologies were obtained through primary screening. Among them, fourteen lactic acid bacterial strains exhibiting stable and potent inhibitory activity against *Salmonella, Escherichia coli,* and *S. aureus* (Figures 1A–D) were shortlisted, with strain D428 selected for subsequent analyses. The colonial morphology and Gram staining characteristics of D428 are depicted in Figures 1E,F. On MRS agar supplemented with CaCO_3_, the D428 strain formed transparent calcium-dissolving halos, producing milky-white, spherical colonies with smooth surfaces and regular margins. Phylogenetic analysis based on 16S rDNA gene sequencing revealed 100% sequence identity to *L. salivarius* strain CPU9601 (MG017448.1), confirming its taxonomic classification (Figure 1G).

### Biological characteristics of *Ligilactobacillus salivarius* D428 strain

#### Growth kinetics

**Figure 2:**
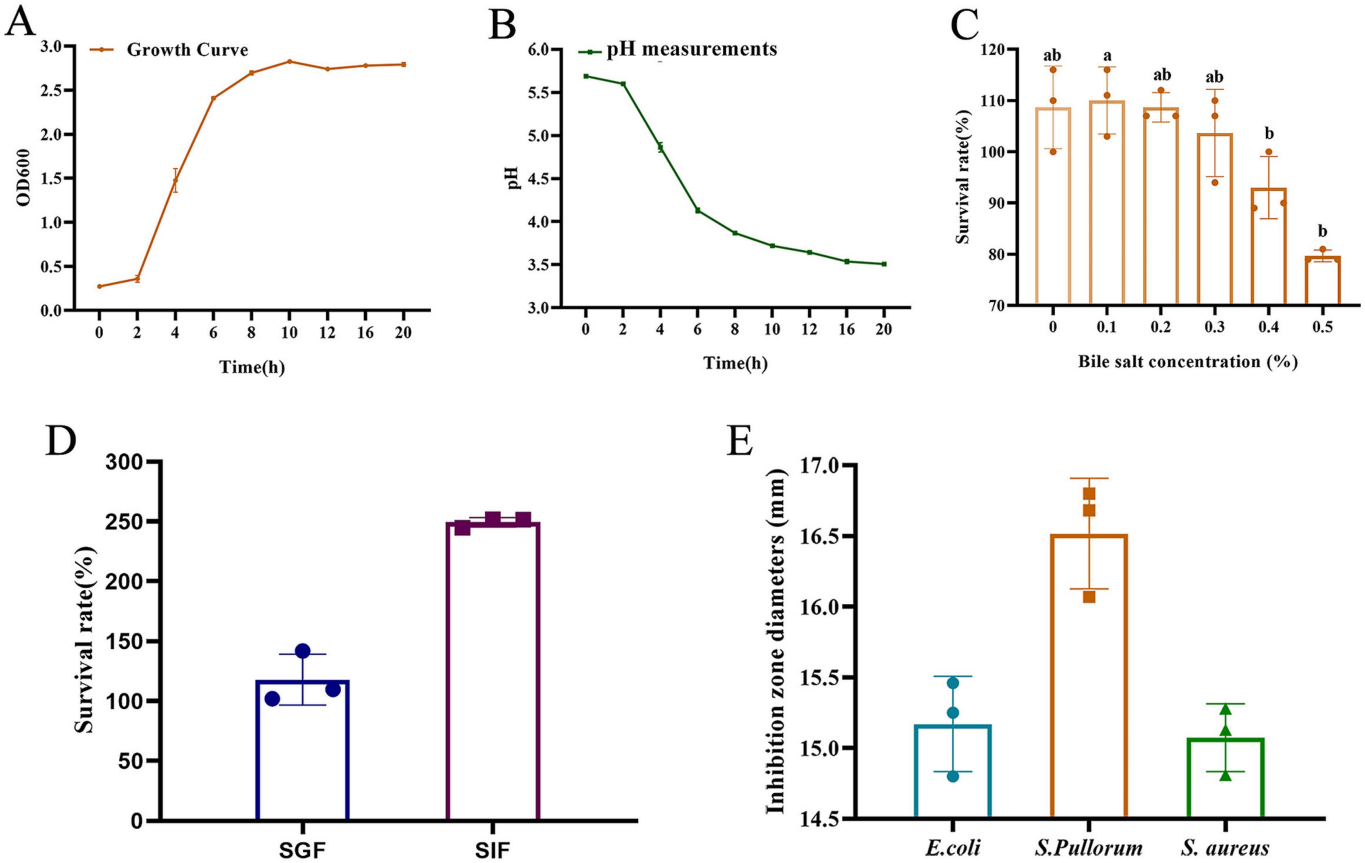
Biological characteristics of the *L. salivarius* D428 strain. **(A)** The growth curve of D428. **(B)** The acid production capacity. **(C)** Bile salt tolerance. **(D)**. Simulated gastric fluid and intestinal fluid tolerance. SGF means simulated gastric fluid, and SIF means simulated intestinal fluid. **(E)** Antibacterial tests. *E. coli* means *Escherichia coli*, *S. pullorum* means *Salmonella pullorum*, and *S. aureus* means *Staphylococcus aureus*. Different letters indicate statistically significant differences (*p* < 0.05).

As presented in Figure 2A, the growth curves of *L. salivarius* strain D428 exhibited a typical sigmoidal pattern: The strain displayed slow growth during the lag phase (0–2 h), entered logarithmic phase with rapid proliferation between 2 and 8 h, and reached stationary phase with steady growth from 8 to 20 h.

#### Acid production capacity

The *L. salivarius* strain D428 exhibited its acid production capacity, evidenced by progressive pH reduction in MRS broth. As shown in Figure 2B, the pH value of the culture gradually decreased with prolonged cultivation time. Minimal pH change occurred during the lag phase (0–2 h), followed by a sharp decline during logarithmic growth (2–8 h), and a gradual decrease throughout the stationary phase (8–20 h).

#### Bile salt tolerance

As shown in Figure 2C, with the increase of bile salt concentration in MRS broth, the survival rates of D428 decreased. At concentrations ≤0.3%, survival rates exceeded 100% with no significant variation. At 0.4% bile salt, the survival rate was 93.00%, whereas increasing the concentration to 0.5% significantly reduced survival to 79.67%, indicating favorable tolerance to bile salts up to 0.5%.

#### Simulated gastric fluid and intestinal fluid tolerance

As shown in Figure 2D, the viable counts of *L. salivarius* strain D428 remained stable after 2 h of incubation in simulated gastric fluid and simulated intestinal fluid, with survival rates exceeding 100%. These results indicate that the D428 strain can tolerate and potentially proliferate under gastrointestinal conditions.

#### *In vitro* antibacterial activity

Using the Oxford cup method, *L. salivarius* strain D428 exhibited significant and differential inhibitory effects against *Salmonella pullorum* (*S. pullorum*), *E. coli*, and *S. aureus*, with the strong activity observed against *S. pullorum* (Figure 2E). MRS broth was the negative control, showing no growth inhibition throughout the experiment.

### ARTP mutagenesis and strain breeding

#### ARTP mutagenesis

**Figure 3:**
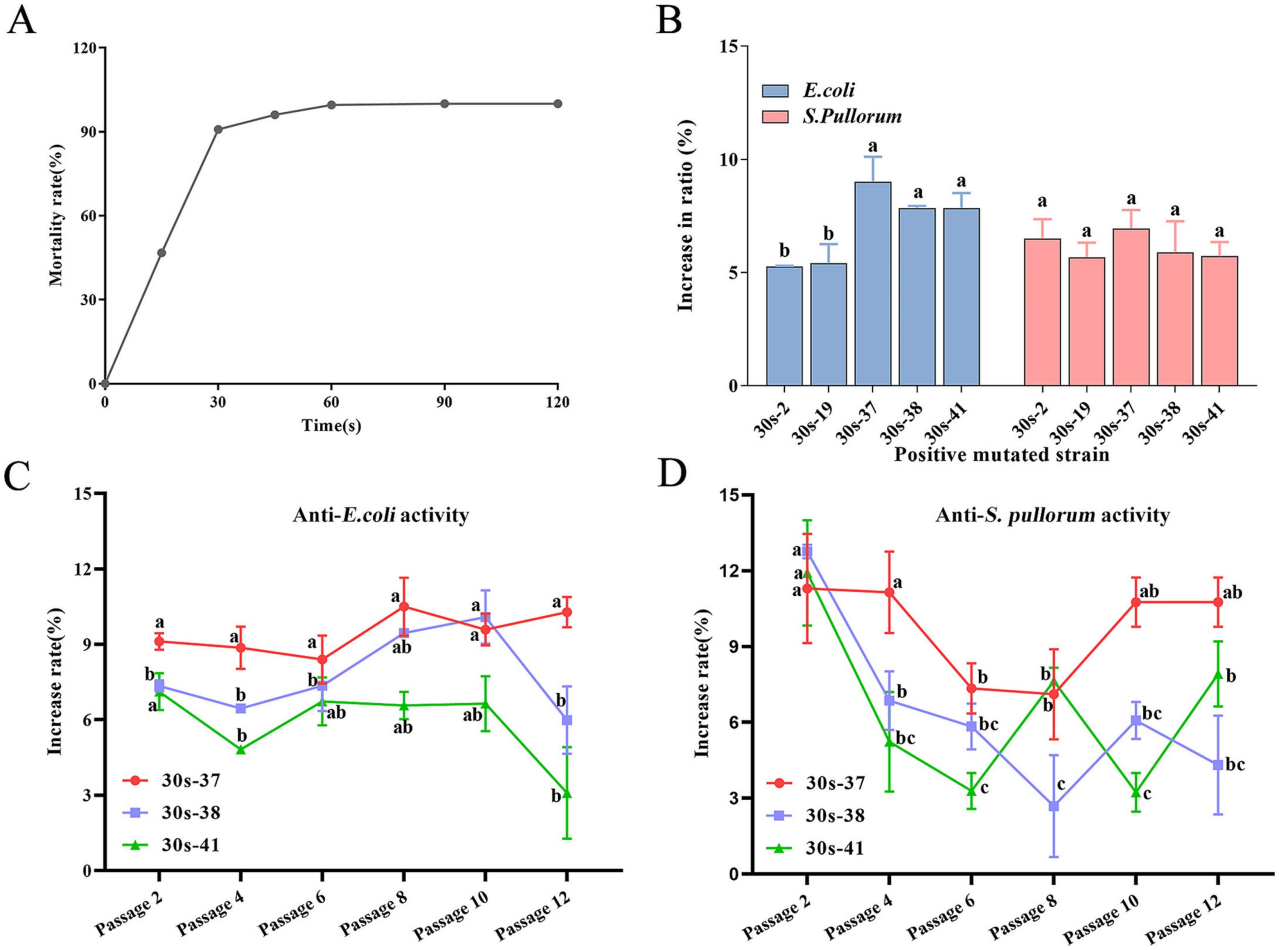
Mutagenesis and breeding of *L. salivarius* D428 by ARTP. **(A)** The lethal curve of *L. salivarius* D428. **(B)** The antibacterial ability of mutant strains against *E. coli* and *S. pullorum*. The statistical analysis was carried out on the differences in the antibacterial ability of different mutant strains against the same pathogenic bacterium. The increase in ratio refers to the proportion by which the antibacterial ability of the mutant strain is enhanced compared to strain D428. **(C,D)** The verification of the stability of antibacterial ability against *E. coli*. And *S. pullorum*, respectively. The increased rate means the change in the antibacterial ability of the strain after mutation compared with that of the D48 strain before mutation. The statistical analysis was carried out on the differences in the antibacterial ability of the same mutant strain across different passages. Different letters in the same line indicate statistically significant differences (*p* < 0.05).

Based on calculations, the lethality curve of *L. salivarius* strain D428 was presented in Figure 3A. The lethality increased monotonically with ARTP mutagenesis time. The lethality was 90.08% at 30 s, 96.08% at 45 s, and 99.57% at 60 s, reaching 100% lethality beyond 90 s.

#### Positive mutation screening

Colonies from 15-, 30-, and 45-s mutagenesis groups were subjected to *in vitro* antibacterial assays. From 389 total colonies across these groups, 185 were randomly selected, yielding 103 mutants with enhanced antibacterial activity compared to the wild-type strain (data not shown). Positive mutation rates at these three time points are summarized in Table 1, with the 30-s group exhibiting the highest mutation frequency.

**Table 1:** The positive mutation rate of D428 induced by ARTP.

Treated time	Picked colonies	Forward mutation colonies	Forward mutation rate
15 s	64	26	40%
30 s	93	62	66.67%
45 s	28	15	53.57%

#### Mutant isolation and characterization

The *L. salivarius* D428 strain was subjected to ARTP mutagenesis for 30 s. From 200 post-mutagenesis colonies, 92 single colonies were randomly selected for subculture and antibacterial activity screening. Following successive subcultures and screening, 18 mutants with enhanced antibacterial activity relative to the wild-type strain were isolated. Among these, five strains exhibited > 5% enhancement in antibacterial activity (Figure 3B), with 30s-37 displaying the highest activity, followed by 30s-41, 30s-38, 30s-2, and 30s-19. Notably, no improvement was observed in anti-*S. aureus* activity across all mutants.

#### Stability and biological consistency

Three top-performing mutants (30s-37, 30s-38, and 30s-41) were selected for passage stability assays, with antibacterial activity assessed every two passages. As shown in Figure 3, mutant 30s-37 exhibited superior anti-*E. coli* (Figure 3C) and anti-*S. pullorum* (Figure 3D) enhancement and stability. Furthermore, no significant differences were observed between 30s-37 and the wild-type D428 regarding growth kinetics, acid production, bile salt tolerance, or artificial gastric and intestinal fluids resistance (Supplementary Figure S3).

### Effects of *Ligilactobacillus salivarius* D428 and 30s-37 on broiler growth performance and cecal microbial community

#### Growth performance analysis

The impacts of *L. salivarius* D428 and 30s-37 on broiler growth performance across different stages are shown in Table 2 below. All groups exhibited consistent initial body weights at the experiment onset. No significant differences were observed during the chick stage (1–25 days) in growth parameters, though the 30s-37 group showed marginal performance advantages. In the middle-stage period (26–45 days), final weights and average daily feed intakes (ADFI) remained comparable among groups. However, the D428 group exhibited a significantly higher average daily gain (ADG) than the control group (*p* < 0.05), and a significantly lower feed conversion ratio (FCR) than both the 30s-37 and control group (*p* < 0.05). The 30s-37 group recorded the lowest mortality and culling rates, significantly lower than the control group (*p* < 0.05). Overall, the D428 group performed better at this stage than the 30s-37 group. In the late-stage (46–71 days), most growth indicators were comparable, except for mortality and culling rates, where D428 showed the highest values, significantly exceeding 30s-37 (*p* < 0.05). Over the entire trial (1–71 days), D428 and 30s-37 increased ADG by 4.4 and 2.4%, respectively, and reduced FCR compared to the control (*p* < 0.05). The 30s-37 group achieved the lowest mortality-culling rate (1.33%), significantly lower than the control (*p* < 0.05).

**Table 2:** Effects of *L. salivarius* D428 and 30s-37 on the growth performance of broiler chickens at different stages.

Monitoring indicators	D428 group	30s-37 group	Control group
1–25 days			
Initial body weight (g/chicken)	33.7 ± 0.0	33.7 ± 0.0	33.7 ± 0.0
Final body weight (g/chicken)	359 ± 7	363 ± 6	356 ± 6
Average daily feed intake (g/day/chicken)	23.5 ± 0.3	23.6 ± 0.5	23.3 ± 0.4
Average daily weight gain (g/day/chicken)	13.0 ± 0.3	13.2 ± 0.2	12.9 ± 0.3
Feed conversion ratio	1.81 ± 0.02	1.79 ± 0.01	1.80 ± 0.01
Mortality and culling rate	1.60 ± 0.50	0.80 ± 0.33	1.87 ± 0.33
46–71 days			
Initial body weight (g)	359 ± 7	363 ± 6	356 ± 6
Final body weight (g)	838 ± 21	799 ± 23	785 ± 27
Average daily feed intake (g/day/chicken)	62.6 ± 1.6	59.8 ± 1.7	60.6 ± 1.6
Average daily weight gain (g/day/chicken)	23.9 ± 0.8^a^	21.8 ± 1.0^ab^	21.4 ± 1.1^b^
Feed conversion ratio	2.62 ± 0.04^b^	2.75 ± 0.06^a^	2.85 ± 0.09^a^
Mortality and culling rate	1.09 ± 0.51^ab^	0.53 ± 0.53^b^	2.17 ± 0.70^a^
46–71 days			
Initial body weight (g)	847 ± 21	812 ± 18	792 ± 26
Final body weight (g)	1,630 ± 36	1,602 ± 32	1,560 ± 49
Average daily feed intake (g/day/chicken)	100.5 ± 2.2	100.2 ± 2.0	99.6 ± 2.6
Average daily weight gain (g/day/chicken)	31.3 ± 1.21	31.6 ± 0.6	30.72 ± 1.26
Feed conversion ratio	3.09 ± 0.07	3.04 ± 0.02	3.12 ± 0.09
Mortality and culling rate	1.14 ± 0.29^a^	0.00 ± 0.00^b^	0.58 ± 0.36^ab^
1–71 days			
Initial body weight (g)	33.7 ± 0.0	33.7 ± 0.0	33.7 ± 0.0
Final body weight (g)	1,630 ± 36	1,602 ± 32	1,560 ± 49
Average daily feed intake (g/day/chicken)	60.8 ± 1.0	59.9 ± 1.2	59.8 ± 1.4
Average daily weight gain (g/day/chicken)	22.7 ± 0.5	22.2 ± 0.5	21.7 ± 0.7
Feed conversion ratio	2.68 ± 0.03^b^	2.70 ± 0.01^b^	2.76 ± 0.04^a^
Mortality and culling rate	3.73 ± 0.88^ab^	1.33 ± 0.42^b^	4.53 ± 0.68^a^

#### Cecal microbial community analysis

**Figure 4:**
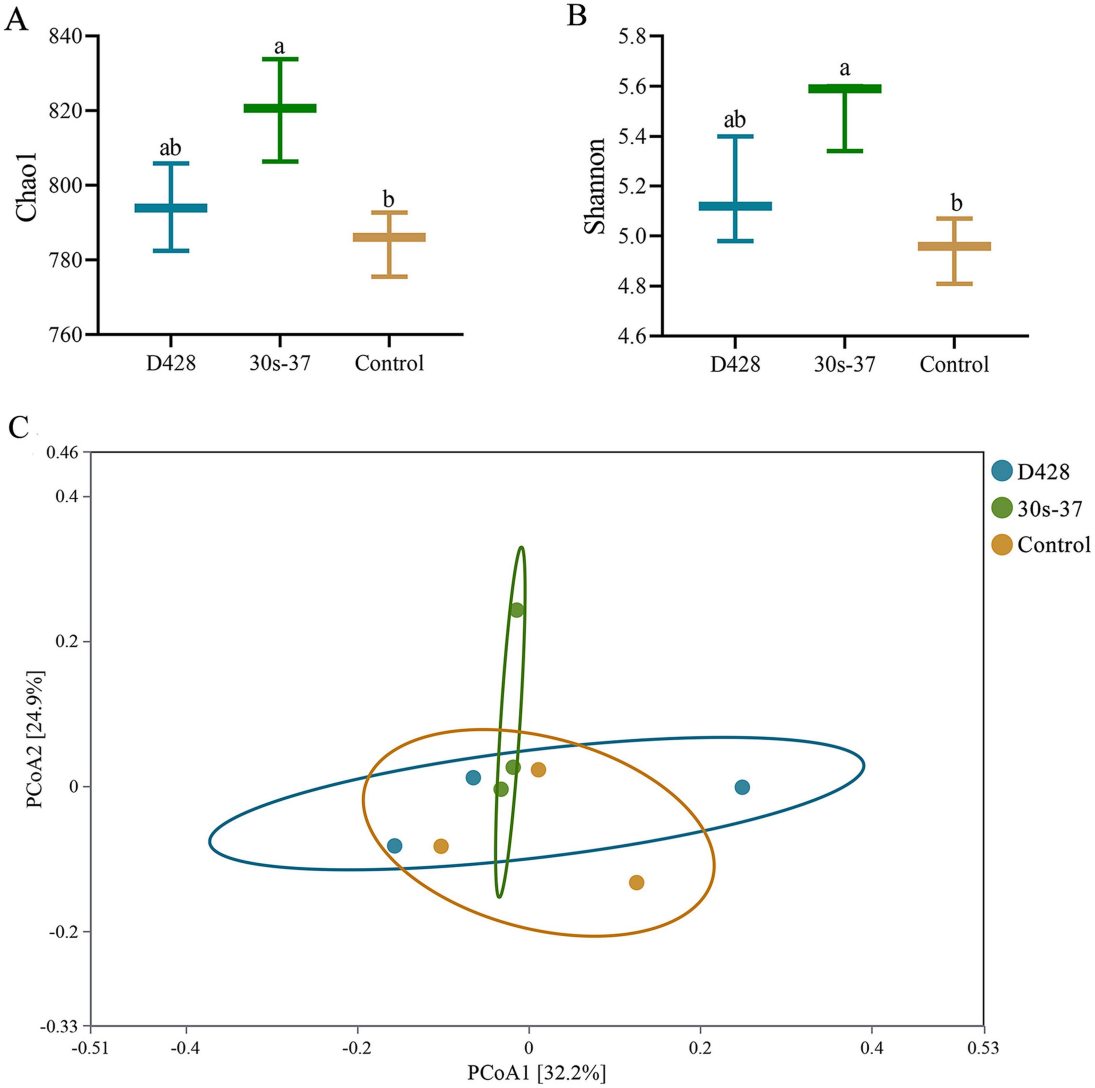
*α*-diversity analysis and PCoA analysis of cecum microorganisms. **(A,B)** Alpha diversity analysis. **(C)** PcoA analysis.

To investigate the mechanisms underlying growth improvements, cecal samples from three randomly selected broilers per group were subjected to microbiota profiling. The 30s-37 group exhibited significantly higher species richness (Chao1 index) and diversity (Shannon index) than the control (Figure 4A), while D428 showed non-significant increases compared to the control. Principal coordinate analysis (PCoA) based on Bray-Curtis distances revealed distinct clustering between the 30s-37 and control groups (Figure 4B), indicating significant microbiota structural divergence. D428 and control samples showed overlapping distributions, suggesting similar community structures.

**Figure 5:**
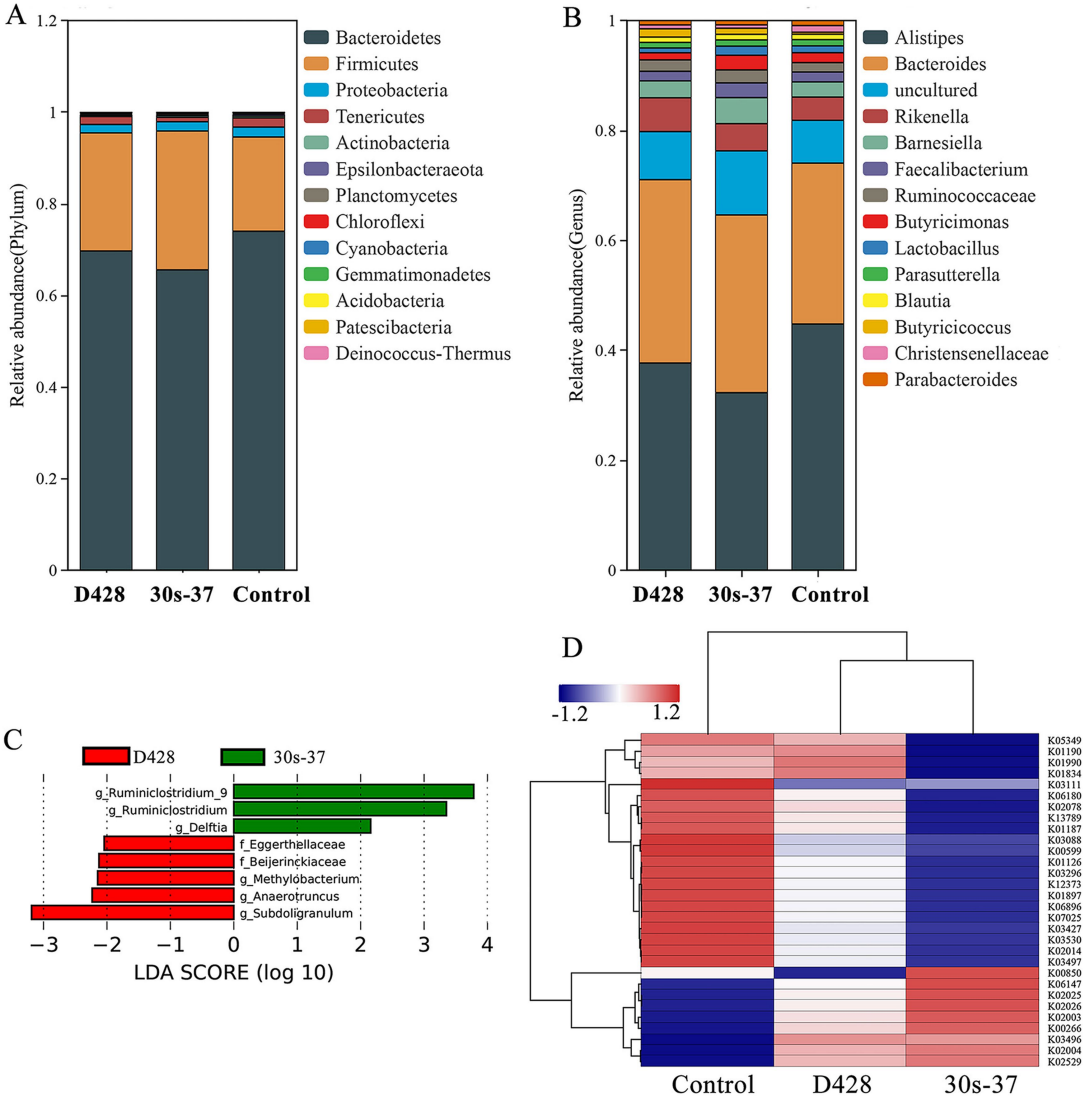
Effect of *L. salivarius* on cecum microorganisms of broilers. **(A)** The relative abundance is based on phylum level. **(B)** The relative abundance is based on genus level. **(C)** The LEfSe analysis. **(D)** KO cluster analysis.

At the phylum level, *Bacteroidetes* and *Firmicutes* were the dominant phyla in all groups. Supplementation with *L. salivarius* significantly reduced *Bacteroidetes* relative abundance (from 73.95% in control to 69.65% in D428 and 65.53% in 30s-37) and increased *Firmicutes* (from 20.57 to 25.78 and 30.30%, respectively), with more pronounced changes in the 30s-37 group (Figure 5A; Supplementary Figure S1). At the genus level, *Alistipes* abundance was significantly reduced in both treatment groups compared to the control, with no differences between D428 and 30s-37 (Figure 5B; Supplementary Figure S2).

**Figure 6:**
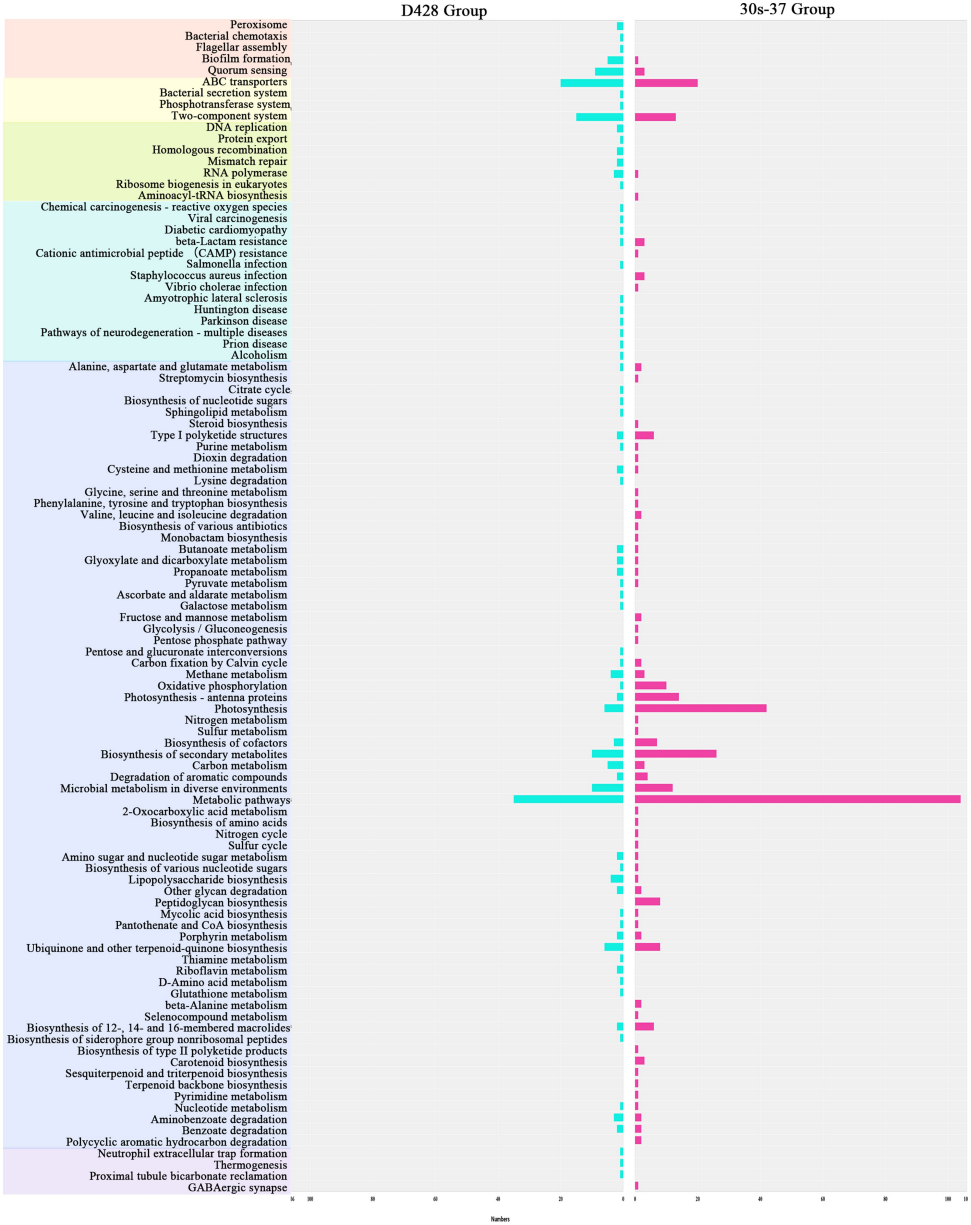
Enrichment of the increased KO pathways after the addition of *L. salivarius*. Different primary KEGG pathways are represented by different colors. The abscissa in the figure represents the number of marker genes.

Linear discriminant analysis effect size (LEfSe) identified distinct biomarker taxa: *Eggerthellaceae*, *Beijerinckiaceae*, *Methylobacterium*, *Anaerotruncus*, and *Subdoligranulum* were enriched in D428, while *Ruminiclostridium* and *Delftia* characterized the 30s-37 group (Figure 5C). Functional annotation through KEGG pathway analysis revealed significant metabolic reprogramming in treatment groups (Figure 5D). D428 upregulated pathways related to bacterial chemotaxis, flagellar assembly, and quorum sensing, whereas 30s-37 exhibited enhanced antimicrobial resistance (e.g., *β*-lactam and cationic antimicrobial peptide resistance) and secondary metabolite biosynthesis (Figure 6). At the KEGG Level 1 pathway, D428 preferentially enriched cellular and genetic information processing pathways, while 30s-37 showed greater increases in metabolic pathways, aligning with its superior growth-promoting phenotype. These findings indicate that *L. salivarius* supplementation modulates cecal microbiota composition and functional pathways, potentially improving nutrient utilization and host resistance, thereby enhancing broiler growth performance and reducing mortality-culling rates.

## Discussion

*Lactobacillus* is a pivotal probiotic that can colonize in the gastrointestinal tract, where it helps maintain intestinal flora homeostasis. Its application in livestock and poultry farming has expanded due to its ability to modulate host microbiota through multiple mechanisms, most notably the “barrier effect,” which involves the competitive exclusion of pathogens by limiting their intestinal colonization (Butel, 2014). *L. salivarius* strains, in particular, exhibit probiotic traits such as bacteriocin secretion, pathogen inhibition, and gut microbiota modulation, contributing to improved broiler growth performance (Xu et al., 2022; Sureshkumar et al., 2021; Sayan et al., 2018). The ideal source of probiotics is the intestinal microbiota from the same category of animals, adapted to the original micro-ecosystem, natural environment, and host (Ozen et al., 2023; Shah et al., 2021). In this study, *L. salivarius* strain D428, which originated from chicken ceca, characterized by bile salt tolerance, artificial gastric and intestinal fluids tolerance, and moderate antibacterial activity, was selected as the parental strain for this study.

Enhancing antibacterial activity through mutagenesis may boost probiotic efficacy by strengthening pathogen inhibition and microbiota regulation. Previous studies have demonstrated ARTP mutagenesis as a powerful tool for strain improvement: Wang *et al.* achieved a 40% increase in avermectin B1a production in *Streptomyces avermitilis* with a 21% positive mutation rate (Wang et al., 2010). Hua *et al*. enhanced salt tolerance and petroleum hydrocarbon degradation in *Enterobacter cloacae* (Hua et al., 2010). Zhao *et al*. improved DHA production by 1.8-fold in a mutant strain (Zhao et al., 2018). Guided by these precedents, we subjected D428 to ARTP mutagenesis, isolating mutant 30s-37, which exhibited the most stable and pronounced antibacterial enhancement, to investigate its effects on broiler growth and cecal microbial communities.

Supplementation with D428 and 30s-37 altered broiler cecal microbiota composition and function, correlating with improved growth performance. While both strains reduced FCR and mortality-culling rates compared to the control, 30s-37 uniquely demonstrated superior early-stage growth promotion. Although growth performance (e.g., body weight gain, FCR) did not differ significantly between 30s-37 and the parental strain D428 across the entire trial, 30s-37 significantly reduced mortality-culling rates (*p* < 0.05), particularly during the chick stage (1–25 days). To investigate underlying mechanisms, cecal microbial community profiling was performed at the trial’s conclusion, revealing that 30s-37 promoted greater cecal microbiota richness (Chao1 index) and diversity (Shannon index) compared to both D428 and the control group.

At the phylum level, *Firmicutes* and *Bacteroidetes* dominated the cecal microbiome in all groups. Supplementation with *L. salivarius* significantly increased *Firmicutes* abundance and decreased *Bacteroidetes*, driving a microbial shift toward a short-chain fatty acid (SCFA)-producing phenotype associated with enhanced intestinal health (Sayan et al., 2018; Ozen et al., 2023; Shah et al., 2021). This shift aligned with findings from Li *et al*. and Wang *et al.*, who reported similar associations between Firmicutes-enriched microbiota and improved host energy metabolism (Wang et al., 2017). *Firmicutes*-derived SCFAs lower intestinal pH, fostering anaerobic probiotic growth and suppressing pathogenic proliferation, while Bacteroidetes primarily mediate macromolecule digestion and energy provision (Wexler, 2007). At the genus level, *Alistipes* was the most abundant taxon across all groups. While *Alistipes* is known to produce acetate via carbohydrate fermentation, its elevated proportion has been linked to intestinal inflammation in some cases (Rautio et al., 2003; Saulnier et al., 2011), precluding definitive conclusions about its probiotic role in this study. LEfSe analysis identified strain-specific biomarker taxa: *Ruminiclostridium*, *Anaerotruncus*, and *Subdoligranulum* were enriched in both treatment groups, contributing to cellulose/protein degradation and SCFA (e.g., butyrate) biosynthesis processes critical for intestinal epithelial cell health and microecological stability (Fosses et al., 2017; Zhang et al., 2021; Liu et al., 2023). Notably, *Anaerotruncus* includes species associated with intestinal infections, highlighting the complex interplay between microbial taxa and host outcomes. *Eggerthellaceae* members, enriched in D428-treated birds, facilitate polyphenol metabolism, producing anti-inflammatory and antioxidant byproducts (Pang et al., 2023), while the roles of *Beijerinckiaceae*, *Methylobacterium*, and *Delftia* remain uncharacterized. These compositional changes suggest that *L. salivarius* supplementation enhances the abundances and diversity of beneficial microbiota, potentially improving nutrient utilization and intestinal barrier function to reduce mortality-culling rates. The differential microbiota regulation between D428 and 30s-37 may arise from their divergent antibacterial profiles, supporting the hypothesis that targeted mutagenesis through enhanced traits such as antibacterial activity can optimize probiotic efficacy via microbiota-mediated mechanisms.

Functional annotation based on 16S rRNA sequencing revealed that *L. salivarius* supplementation significantly altered cecal microbiota metabolic profiles, predominantly enriching pathways related to energy and nutrient metabolism. KEGG Level 1 pathway analysis showed pronounced enrichment in Metabolism, Genetic Information Processing, and Environmental Information Processing, with the Metabolism category exhibiting the highest level of enrichment. Comparative analysis between D428 and 30s-37 highlighted strain-specific functional divergence: 30s-37 was significantly enriched in antimicrobial resistance and biosynthesis pathways, including β-lactam resistance, cationic antimicrobial peptide (CAMP) resistance, *S. aureus* infection, secondary metabolite biosynthesis, and peptidoglycan biosynthesis. These findings align with the targeted enhancement of antibacterial activity in the mutagenized strain, demonstrating successful functional adaptation to the intestinal environment. Mechanistically, both strains likely improve broiler growth performance by optimizing metabolic pathways for nutrient utilization, while 30s-37 further strengthens disease resistance through enhanced antimicrobial biosynthesis and microbiota-structuring effects. These functional shifts support the hypothesis that ARTP mutagenesis-driven antibacterial improvements prime probiotics to modulate intestinal microecology, thereby reducing mortality-culling rates through dual mechanisms of direct pathogen inhibition and metabolic environment optimization.

## Conclusion

This study demonstrates that ARTP mutagenesis effectively enhances *L. salivarius* probiotic properties by improving antibacterial activity, leading to altered cecal microbiota composition and metabolic pathway enrichment in broilers. The mutant strain 30s-37 represents a promising candidate for poultry applications, illustrating that targeted *in vitro* mutagenesis is a viable strategy to engineer probiotics with enhanced microbiota-regulating capabilities.

## Data Availability

The datasets presented in this study can be found in online repositories. The names of the repository/repositories and accession number(s) can be found at: https://www.ncbi.nlm.nih.gov/, PRJNA1152287.

## Supplementary material

SUPPLEMENTARY FIGURE S1 Biological characteristics of *L. salivarius* D428 strain and 30s-37 strain. **(A)** The growth curve of D428 and 30s-37 strains. **(B)** The acid production capacity. **(C)** Bile salt tolerance. **(D)** Simulated gastric fluid and intestinal fluid tolerance. SGF means simulated gastric fluid, and SIF means simulated intestinal fluid.

SUPPLEMENTARY FIGURE S2 The relative abundance of cecum microorganisms of broilers on phylum level. The statistical significance was used to analyze the differences in the relative abundance of cecum microorganisms among different groups. Different letters indicate statistically significant differences in the results (*p* < 0.05).

SUPPLEMENTARY FIGURE S3 The relative abundance of cecum microorganisms of broilers on genus level. The statistical significance was used to analyze the differences in the relative abundance of cecum microorganisms among different groups. No significant difference between the two groups with the same letters. Different letters indicate statistically significant differences in the results (*p* < 0.05).
