# Supplementary figures and images for: Enhancing the antibacterial ability of Ligilactobacillus salivarius through ARTP mutagenesis breeding: an effective strategy to improve its probiotic properties
Source: Front Microbiol. 2025 Jul 21;16:1595651. doi: 10.3389/fmicb.2025.1595651 (PMC12318943; doi:10.3389/fmicb.2025.1595651)

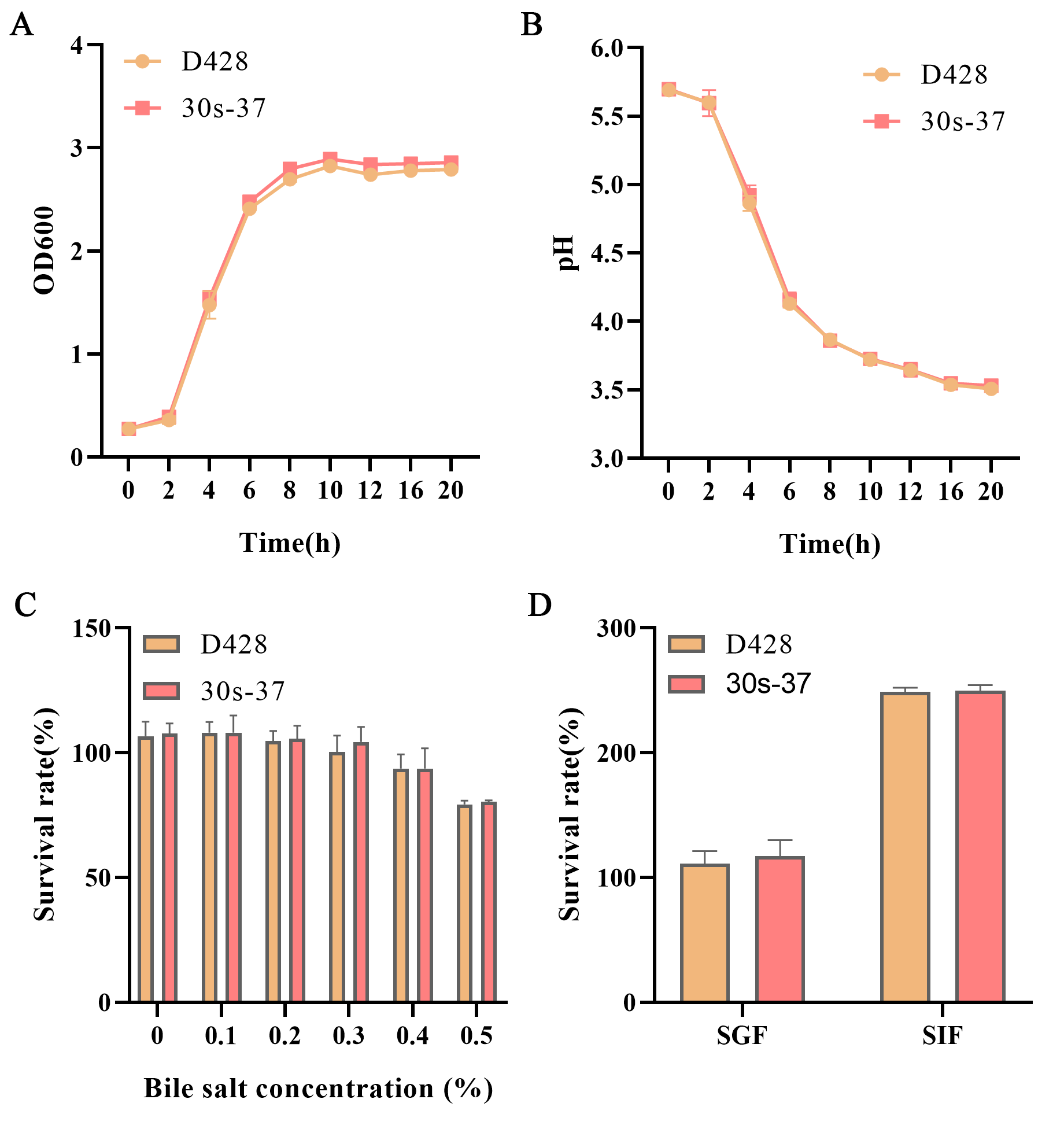

Supplement: SUPPLEMENTARY FIGURE S1 — Biological characteristics of L. salivarius D428 strain and 30s-37 strain. (A) The growth curve of D428 and 30s-37 strains. (B) The acid production capacity. (C) Bile salt tolerance. (D) Simulated gastric fluid and intestinal fluid tolerance. SGF means simulated gastric fluid, and SIF means simulated intestinal fluid. [file Image_1.tif]

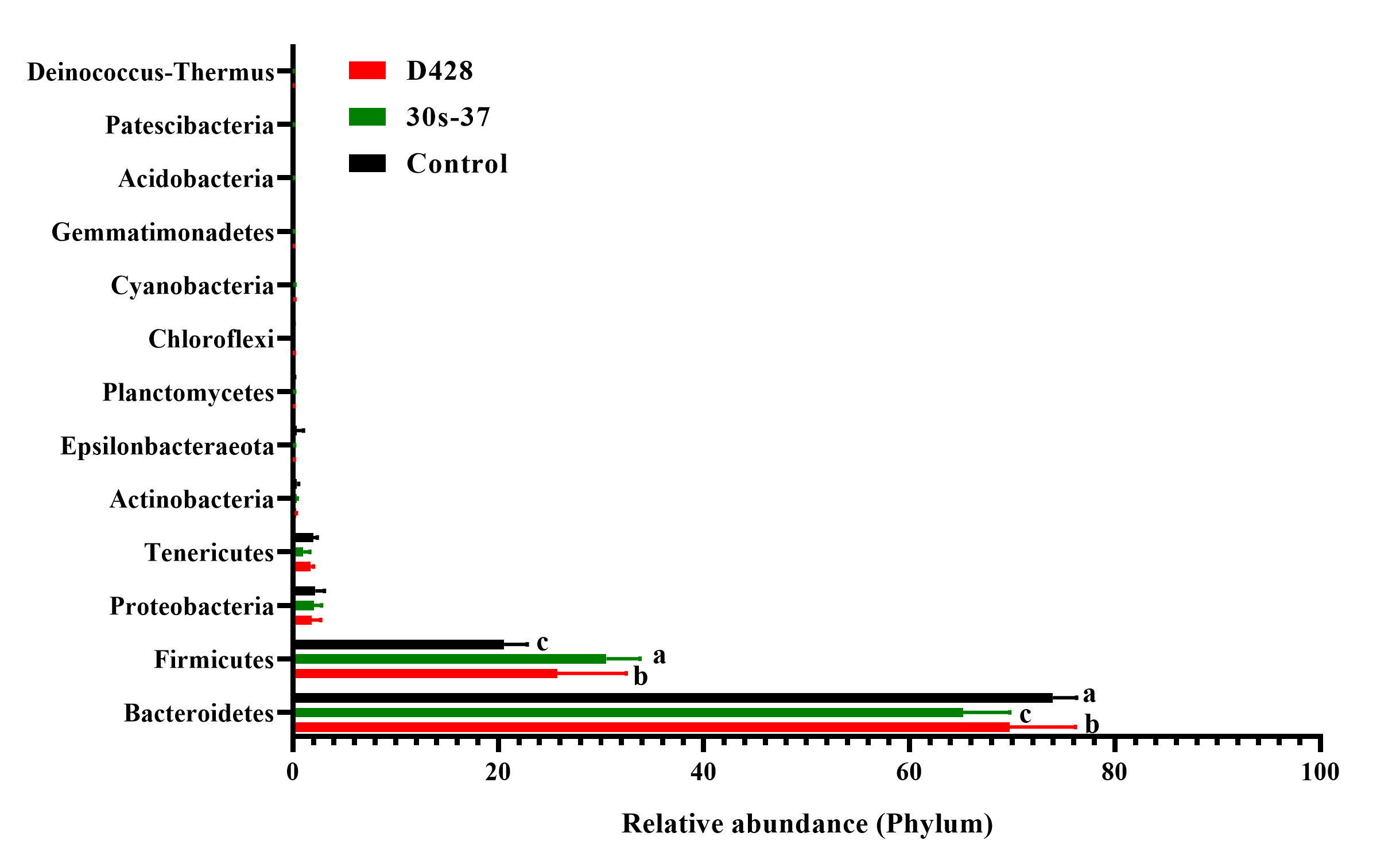

Supplement: SUPPLEMENTARY FIGURE S2 — The relative abundance of cecum microorganisms of broilers on phylum level. The statistical significance was used to analyze the differences in the relative abundance of cecum microorganisms among different groups. Different letters indicate statistically significant differences in the results (p < 0.05). [file Image_2.tif]

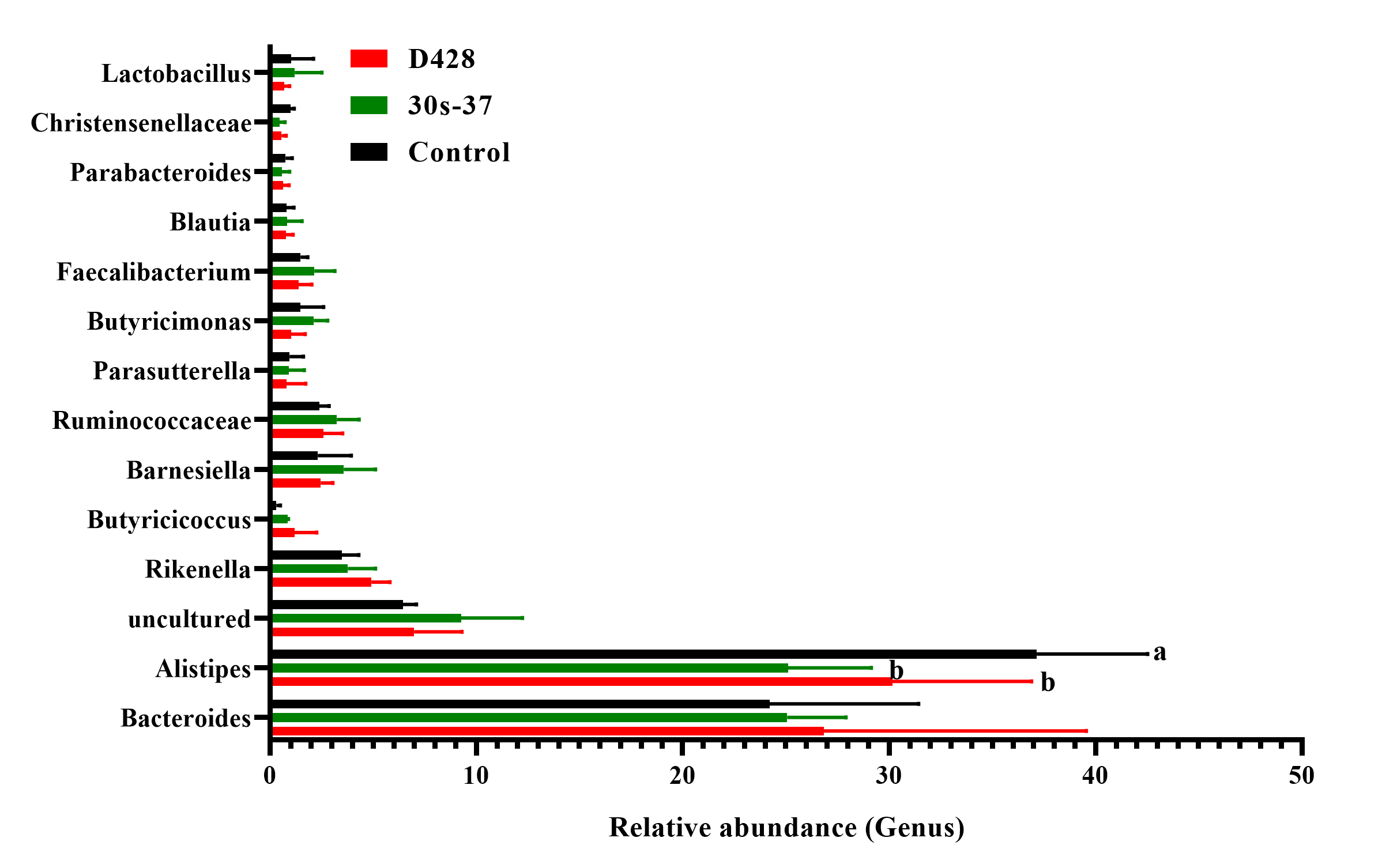

Supplement: SUPPLEMENTARY FIGURE S3 — The relative abundance of cecum microorganisms of broilers on genus level. The statistical significance was used to analyze the differences in the relative abundance of cecum microorganisms among different groups. No significant difference between the two groups with the same letters. Different letters indicate statistically significant differences in the results (p < 0.05). [file Image_3.tif]
